# Delayed Temporal Lobe Hemorrhage After Initiation of Acyclovir in an Immunocompetent Patient with Herpes Simplex Virus-2 Encephalitis: A Case Report

**DOI:** 10.7759/cureus.980

**Published:** 2017-01-15

**Authors:** Kyle Mueller, Joshua E Ryan, Alex Tai, Rocco A Armonda

**Affiliations:** 1 Neurosurgery, Medstar Georgetown University Hospital

**Keywords:** intracerebral hemorrhage, herpes encephalitis, acyclovir, decompressive hemicraniectomy, lobectomy

## Abstract

Herpes simplex virus (HSV) is the most common cause of non-epidemic, sporadic, acute focal encephalitis in the United States. Inflammation of the vasculature makes them friable and susceptible to hemorrhage. Massive hemorrhage, though rare, can present in a delayed fashion after initiation of acyclovir and often requires surgical intervention. We report a unique case of delayed temporal lobe hemorrhage after initiation of acyclovir in an immunocompetent patient, specifically for its presentation, virology, and surgical management. A 40-year-old left-handed Caucasian female with chronic headaches, along with a 20-pack-year smoking history, presented to an outside facility with one week of diffuse, generalized headache, fever, nausea, and vomiting. Initial cranial imaging was negative for hemorrhage. Cerebrospinal fluid (CSF) studies showed a lymphocytic pleocytosis with elevated protein, along with polymerase chain reaction (PCR) positive staining for HSV, establishing the diagnosis of HSV-2 encephalitis, which is less common in adults. Acyclovir was initiated and one week later while still hospitalized, the patient developed acute altered mental status with cranial imaging showing a large right temporal lobe hemorrhage with significant midline shift. She was transferred to our facility for surgical intervention. Computed tomography angiography (CTA) was negative for any underlying vascular lesion. She was taken to the operating room for a decompressive unilateral (right) hemicraniectomy and temporal lobectomy. She had no postoperative complications and completed a three-week course of acyclovir. She was discharged to acute rehab with plans to return at a later date for cranioplasty. Intracerebral hemorrhage is an uncommon, although possible sequela, of herpes encephalitis. Despite initiation of early antiviral therapy, close monitoring is warranted, given the pathophysiology of the vasculature. Any decline in the neurological exam and/or increasing symptomatology of increased intracranial pressure mandates immediate cranial imaging to evaluate for possible hemorrhage. Emergent surgical intervention is warranted with large temporal lobe hemorrhages.

## Introduction

Herpes simplex virus (HSV) is the most common cause of non-epidemic, sporadic, acute focal encephalitis in the United States [1-3]. Inflammation of the vasculature makes them friable and susceptible to hemorrhage. Massive hemorrhage, though rare, can present in a delayed fashion after the initiation of acyclovir and often requires surgical intervention. Few cases have been reported in the literature with herpes simplex virus, Type 2 (HSV-2) as the viral agent associated with hemorrhage [4-7]. We report a unique case of delayed temporal lobe hemorrhage after initiation of acyclovir in an immunocompetent patient with HSV-2 encephalitis, specifically for its presentation, virology, and surgical management.

## Case presentation

### History and examination

A 40-year-old left-handed Caucasian female with chronic headaches, along with a 20-pack-year smoking history, presented to an outside facility with one week of diffuse, generalized headache, fever, nausea, emesis, and photophobia. Her physical exam was without any focal neurological deficit. Initial cranial imaging was negative for hemorrhage. A lumbar puncture was performed and cerebrospinal fluid (CSF) studies demonstrated 558 white blood cells/mm3 with 100% monocytes, protein 115 mg/dL, and glucose 55 mg/dL. Polymerase chain reaction (PCR) of the CSF was positive for HSV-2. Taken together, the lymphocytic pleocytosis with elevated protein, along with CSF-PCR-positive staining for HSV, established the diagnosis of HSV-2 encephalitis.

**Figure 1:**
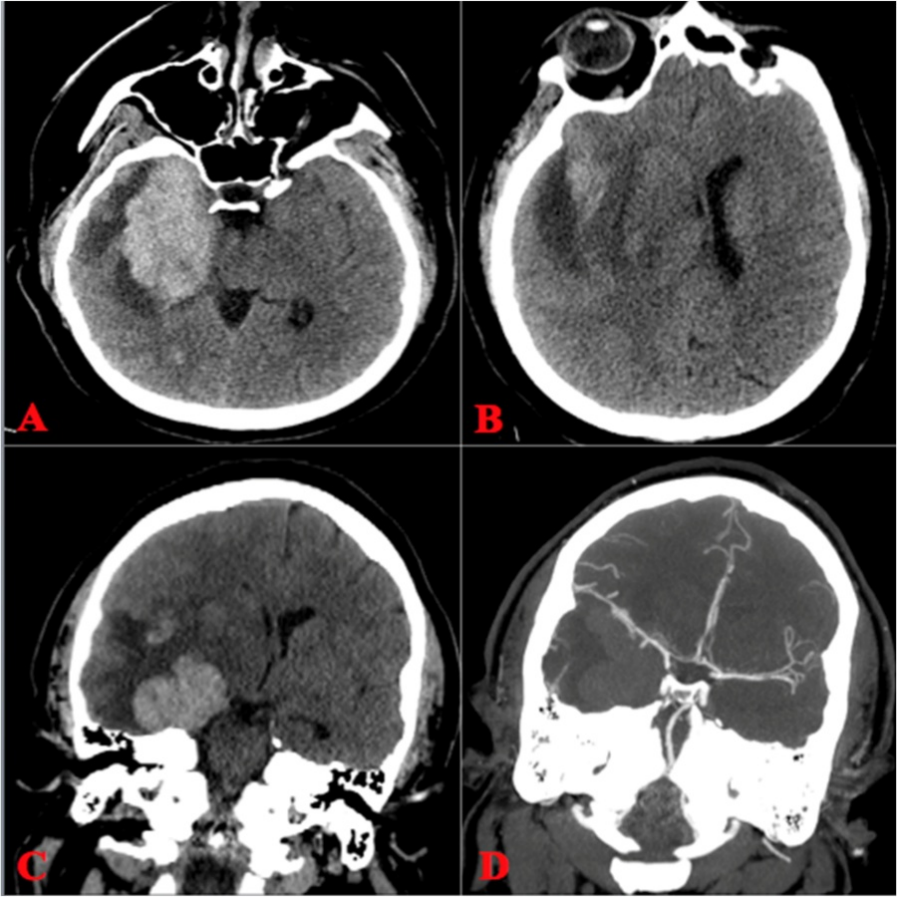
CT and CTA Prior to Surgery A, B) Axial non-contrast head CT demonstrating a large right temporal ICH with mass effect and shift from right to left. C, D) Coronal non-contrast and CTA illustrating no vascular lesion along with elevation in the middle cerebral artery compared to the contralateral side. ICH: intracranial hemorrhage; CTA: computed tomography angiography

Acyclovir was initiated, and one week later while still hospitalized, she developed an acute altered mental status and left-sided weakness with cranial imaging showing a large right temporal lobe hemorrhage with significant midline shift. She was transferred to our facility where a computed tomography angiography (CTA), performed on arrival and shown in Figure 1, was negative for any underlying vascular lesion. There was again the demonstration of a large right temporal lobe intracerebral hemorrhage that was displacing the right middle cerebral artery superiorly as well as midline shift. Informed patient consent was waived due to her condition upon admission.

### Hospital course

**Figure 2:**
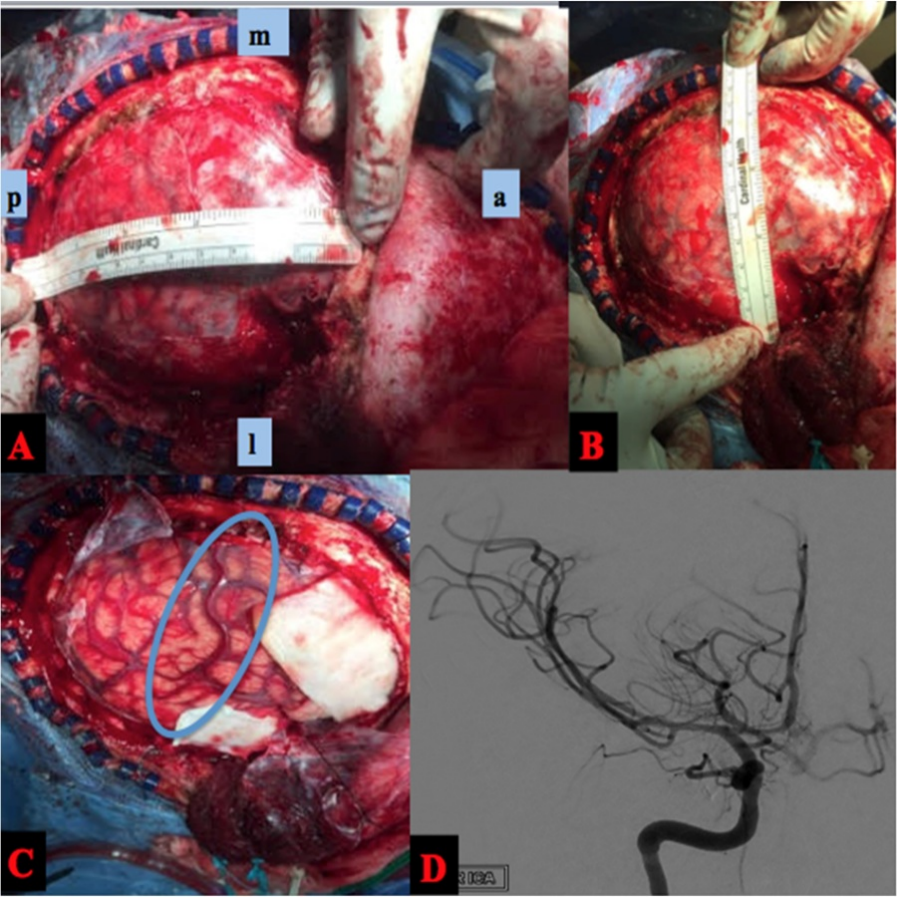
Intraoperative and Postoperative Angiogram A, B) Intraop photos showing measurement of hemicraniectomy dimensions. C) After durotomy was performed, a concerning superficial vein that appeared arterialized was noted. This ended up being due to increased ICP. D) Postoperative angiogram (AP right ICA injection) again showing no vascular lesion along with improvement in the angle of the middle cerebral artery after clot evacuation. AP: anteroposterior; ICA: internal carotid artery

She was taken to the operating room for a decompressive unilateral (right) hemicraniectomy and temporal lobectomy. We had the operating room prepared for a possible vascular lesion despite the initial negative study. Figure 2A-C demonstrates intraoperative images. A 15 x 12 cm (AP x lateral) craniectomy was measured out and performed, ensuring to stay within 2 cm off of the midline to avoid superior sagittal sinus bleeding [8]. The dura was opened in a cruciate fashion. A large red vein was noted superficially and coursed anterior-inferiorly toward the Sylvian fissure. A right temporal lobectomy was performed, measuring 5 cm posterior to the temporal tip. The parenchyma was noted to be very friable. The hematoma was removed, and there were no intraoperative complications. As the brain was decompressed, the red superficial vein normalized to the typical blue color commonly encountered. A postoperative angiogram was performed and demonstrated no vascular lesion as well as a normal alignment of the middle cerebral artery as is seen in Figure 2D.

**Figure 3:**
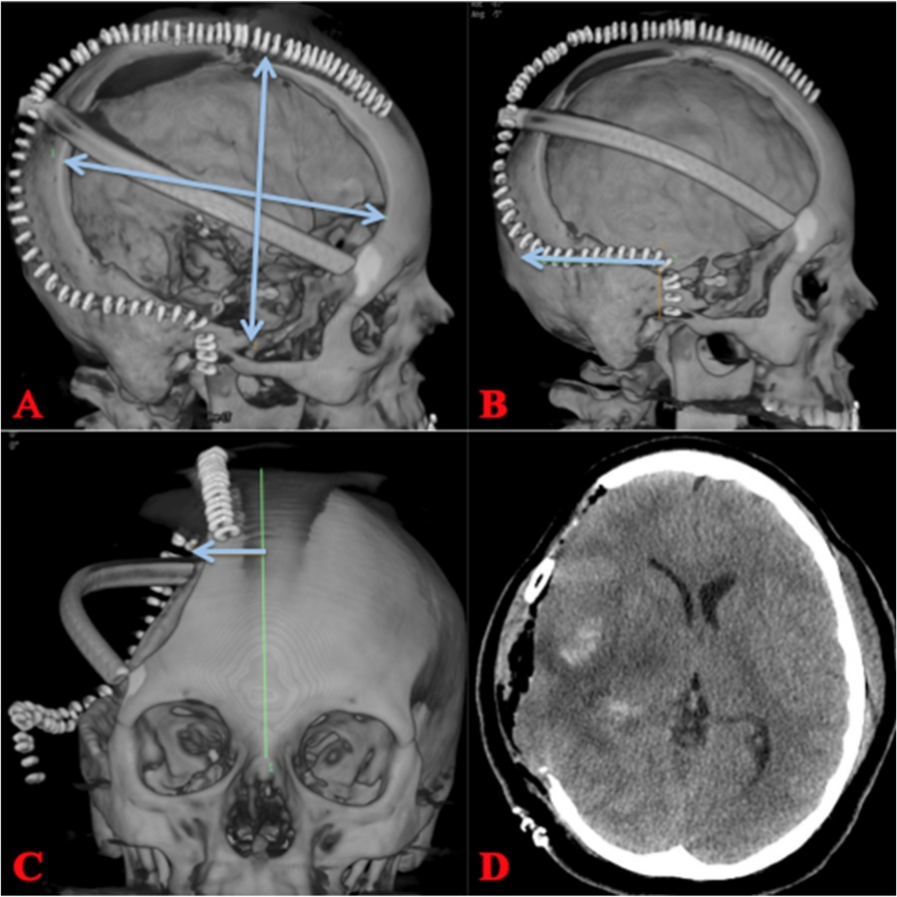
Postoperative Hemicraniectomy A, B) A large right hemicraniectomy with dimensions that should be 15 X 12 cm (AP & lateral). The incision should be carried posteriorly for 6 cm from the temporal root of zygoma. C) Incision should be carried to within a couple centimeters from midline to avoid the superior sagittal sinus. D) Postoperative CT showing craniectomy defect and improvement in mass effect. AP: anteroposterior; CT: computed tomography

Postoperative imaging is shown in Figure 3, which demonstrates good decompression and highlights the appropriate size of the craniectomy.

**Figure 4:**
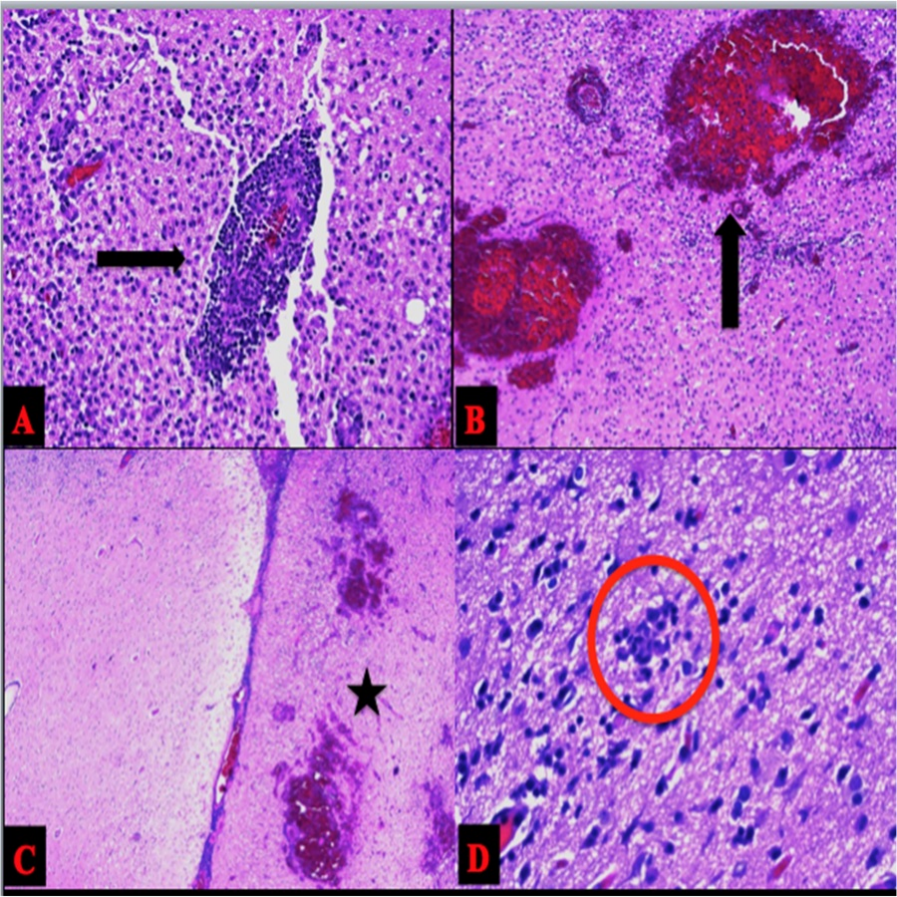
Microscopic Analysis Hematoxylin and eosin stains demonstrating A) Perivascular lymphocytic infiltrate B) Intraparenchymal hemorrhage C) Histiocytic infiltrate on the right.  Normal on the left. Sulcus in the middle. D) Neuronophagia.

Histopathological studies are shown in Figure 4 and are consistent with herpes encephalitis, showing perivascular lymphocytic infiltrate, neuronophagia, and hemorrhage.

She had no postoperative complications and completed a three-week course of acyclovir. She was discharged to acute rehab with plans to return at a later date for cranioplasty.

## Discussion

Herpes encephalitis is a common cause of sporadic, fatal encephalitis. The virus has two types, herpes simplex virus Type 1 (HSV-1) and HSV-2. In adults, HSV-1 is found predominantly in the oral area, and HSV-2 is commonly found in the genital area. HSV-2 is also the predominant type found in neonates. The majority of herpes encephalitis in adults is HSV-1, but a few reports have shown that HSV-2 can also be present [3]. Our case demonstrates a unique aspect of the herpes virology, that HSV-2 encephalitis in adults is exceedingly rare. The hemorrhagic association with the subtypes has not been fully characterized. The mode of transmission for HSV-2 is usually through the birth canal in a mother with active genital herpes, and HSV-1 is thought to possibly involve latent reactivation [8-10]. There appears to be no relationship with the immunocompetent status of a patient and the risk for developing HSV-1, although the relation with HSV-2 is less clear [2]. The use of immunosuppressive agents has been shown to make some patients susceptible to the development of HSV-1, and further research is warranted.

Intracerebral hemorrhage is an uncommon, although possible, sequela of herpes encephalitis. Herpes encephalitis most commonly occurs in the temporal lobes. However, in a retrospective study of 20 patients with herpes simplex encephalitis, Wasay, et al. reported occurrence in extratemporal locations in 11 of the 20 patients (55%) [7]. Hemorrhage can be seen in the area of infection in up to 35% of patients upon admission [6]. The standard of care for treatment involves the rapid initiation of antiviral therapy. Despite initiation of early antiviral therapy, a close neurological assessment is necessary, as there have been rare reports of patients declining after treatment [4]. In our patient, the sudden change in the neurological exam was concerning for an acute intracranial process. The importance of close monitoring of the neurological exam, along with a low threshold for cranial imaging with changes or persistent symptoms, cannot be overstated.

Emergent surgical intervention is often warranted with large temporal lobe hemorrhages because of the need to prevent uncal herniation. Although a diagnosis of herpes encephalitis had been obtained in our patient, preoperative attention to possible vascular etiologies is prudent for the surgeon. Performing a large decompression in a timely fashion can result in improved neurological outcomes for patients. Very large increases in intracranial pressure from the temporal hemorrhage perhaps caused a pseudo-arterialization of the superficial veins that improved as the pressure was lowered.

## Conclusions

HSV-2 encephalitis associated with temporal lobe hemorrhage is rare in the adult population. For patients with the diagnosis of HSV encephalitis, prompt initiation of antiviral therapy is necessary. Any decline in the neurological exam and/or increasing symptomatology of increased intracranial pressure mandates immediate cranial imaging to evaluate for possible hemorrhage. Delayed hemorrhage after prompt initiation of antiviral therapy is a possible sequela. 
